# EVEREST study report 3: diagnostic challenges of polypoidal choroidal vasculopathy. Lessons learnt from screening failures in the EVEREST study

**DOI:** 10.1007/s00417-016-3333-y

**Published:** 2016-05-03

**Authors:** Colin S. Tan, Wei Kiong Ngo, Louis W. Lim, Nikolle W. Tan, Tock H. Lim, on behalf of the EVEREST Study Group

**Affiliations:** 1 Fundus Image Reading Centre, National Healthcare Group Eye Institute, Singapore, Singapore; 2 National Healthcare Group Eye Institute, Tan Tock Seng Hospital, 11 Jalan Tan Tock Seng, Singapore, 308433 Singapore

**Keywords:** EVEREST study, Polypoidal choroidal vasculopathy, Indocyanine green angiography, non-PCV, Age-related macular degeneration

## Abstract

**Purpose:**

To describe screening failures in the EVEREST study by examining the imaging characteristics that enabled differentiation of polypoidal choroidal vasculopathy (PCV) from cases that were subsequently diagnosed not to be PCV.

**Methods:**

Post-hoc analysis of 34 patients with PCV reported as screening failures from EVEREST study. Standardised confocal scanning laser indocyanine green angiography (ICGA) images were graded by the Central Reading Centre to confirm PCV diagnosis based on the presence of early focal sub-retinal hyperfluorescence on ICGA and at least one of the following six diagnostic criteria: (1) nodular appearance of polyp(s) on stereoscopic examination, (2) hypofluorescent halo around nodule(s), (3) presence of a branching vascular network, (4) pulsation of polyp(s) on dynamic ICGA, (5) orange sub-retinal nodules on colour fundus photography, or (6) massive sub-macular haemorrhage (≥4 disc areas in size). Additional detailed image grading was performed with stereo-imaging and dynamic early-phase ICGA.

**Results:**

Of the 95 screened PCV cases, 34 were excluded: (1) cases not suitable for recruitment as per the study protocol (*n* = 14), (2) equivocal lesions on ICGA characterised by small hyperfluorescent dots (*n* = 9), and (3) cases that were definitely not PCV (non-PCV, *n* = 11), identified by definitive diagnoses which included one case each of micro-aneurysm, retinal angiomatous proliferation, retino-choroidal anastomosis, small type-2 choroidal neovascularisation, retinal pigment epithelial (RPE) window defect and disciform scar; two cases of lesions where the choroidal vessel changed its course; and three cases of late-onset RPE staining.

**Conclusions:**

Standardised image grading techniques used in EVEREST study enabled effective differentiation of non-PCV from actual PCV.

**Electronic supplementary material:**

The online version of this article (doi:10.1007/s00417-016-3333-y) contains supplementary material, which is available to authorized users.

## Introduction

Polypoidal choroidal vasculopathy (PCV) is generally considered a subtype of neovascular age-related macular degeneration (nAMD), a variant of Type 1 choroidal neovascularisation (CNV). However, some investigators believe that PCV is a discrete vascular abnormality of the choroidal vessels [1, 2]. PCV mostly affects patients aged 50 to 65 years [3] and accounts for up to 23.9 –54.7 % of presumed nAMD in Asian populations [4–8] and about 8–13 % in Caucasians [9].

The understanding of PCV has evolved considerably over the past few decades [10]. The natural course of PCV has been shown to be favourable compared to typical nAMD, with up to 50 % of patients showing spontaneous resolution of presenting features [11]. However, some patients with PCV may have repeated episodes of haemorrhage and leakage, resulting in significant loss of vision [7, 11, 12]. Typical presentation of eyes with PCV includes serosanguineous maculopathy, haemorrhagic pigment epithelial detachment (PED) and notched serous PED [1]. Orange-red sub-retinal nodules are also frequently seen (in 55.7 % of patients in the EVEREST study [NCT00674323] and 37.4 % in a single-center cohort study). Some patients may sometimes be asymptomatic [13]. In addition, massive sub-macular haemorrhages have also been observed in over 7.5 to 13.1 % of patients, which may lead to a poor visual prognosis [13–15].

The pathophysiology of PCV is still widely debated, particularly whether it is a distinct type of choroidal vasculopathy, or a variant of nAMD, but with some distinct clinical features [16, 17]. PCV appears to have better visual prognosis compared to typical nAMD. Treatment response of PCV appears to differ from typical nAMD. PCV has been reported in some studies to respond better to verteporfin photodynamic therapy (PDT) compared to typical nAMD. The EVEREST study [18] reported a higher rate of polyp closure among patients treated with PDT combined with intravitreal ranibizumab (Novartis AG, Basel, Switzerland) or with PDT monotherapy compared to the group treated with ranibizumab monotherapy. Some authors have reported cases of nAMD resistant to anti-VEGF monotherapy that were later diagnosed to be PCV [19]. However, other studies have also shown that patients with PCV may respond well to treatment with ranibizumab or aflibercept monotherapy [20–22]. In the LAPTOP study, Oishi reported that patients receiving intravitral ranibizumab had better visual outcomes compared to those who received PDT [20, 21]. In a study of intravitreal aflibercept (Bayer AG, Leverkusen, Germany) for patients with PCV, visual acuity improved from 0.31 at baseline to 0.17 at 12 months, with 24.4 % of eyes showing an improvement in visual acuity, compared to 4.5 % who experienced a decrease [23]. While the optimal treatment regimen for PCV remains a matter of discussion, given its differences from typical nAMD, early identification and accurate diagnosis of PCV may result in better management of the disease.

The current evidence-based guidelines provide practical guidance on the clinical diagnosis and management of PCV [24]. Indocyanine green angiography (ICGA) is presently considered an essential diagnostic test for PCV. The characteristic features of PCV as seen on ICGA include typical sub-retinal hyperfluorescent nodules with or without branching vascular network (BVN). Many studies, however, often do not clearly define the meaning of these terms, and it seems likely that authors may be using the same terms to refer to different anatomical abnormalities. Moreover, focal hyperfluorescence seen on ICGA may not always represent PCV lesions. Conditions such as stage 1 retinal angiomatous proliferation (RAP), retinal micro or macro-aneurysm, focal retinal pigment epithelium (RPE) defect and changes in the course of the choroidal vessel often manifest with focal ICGA hyperfluorescence and may be misdiagnosed as PCV [10]. We propose referring to these conditions as “non-PCV”.

Misdiagnosis of these conditions as PCV may result in inappropriate or unnecessary treatment, highlighting the importance of accurate identification and differentiation of PCV from non-PCV. This is also important in randomised clinical trials to ensure inclusion of only cases that meet the study definition of PCV.

EVEREST was the first ICGA-guided, phase IV, multicentre, randomised clinical trial on PCV treatment. A definitive diagnosis of PCV was made by the use of well-defined criteria agreed upon by the EVEREST Study Group, and by the engagement of a Central Reading Centre (CRC) that provided standardised confirmatory diagnosis [13, 18]. Recent reports from the EVEREST study provide detailed descriptions of ICGA imaging standards, grading methods, and diagnostic criteria for PCV [13].

In the past, reports have focused on PCV masquerading as other conditions such as nAMD and central serous chorioretinopathy [25, 26]. However, none to date have focused on the reverse: non-PCV conditions masquerading as PCV. A key knowledge gap is the characteristic of patients with conditions that may mimic PCV in their clinical presentation or imaging features.

In this post-hoc analysis, we describe the angiographic findings of the non-PCV cases in the EVEREST study [18], and highlight the differentiating features.

## Methods

EVEREST was a Phase IV, multicenter, randomised, active controlled, double-masked, exploratory study. The study design and outcomes of the EVEREST study have been published in detail elsewhere [18]. Patients with best corrected visual acuity (BCVA) letter score of 73 to 24 using Early Treatment of Diabetic Retinopathy Study charts at a starting distance of 4 m (20/40 to 20/320 Snellen equivalent) were included. Patients were randomised in a 1:1:1 ratio into three treatment arms: 1) PDT with verteporfin (Novartis AG, Basel, Switzerland) combined with intravitreal ranibizumab, 2) PDT monotherapy, or 3) ranibizumab monotherapy. Standardised imaging protocols, including dynamic and stereo still ICGA (Heidelberg Retinal Angiography (HRA), HRA-C/HRA2/HRA Spectralis, Heidelberg Engineering, Heidelberg, Germany) were used on all patients [13]. Before randomisation, eligibility of the study eye was confirmed by the CRC (Fundus Image Reading Centre, National Healthcare Group Eye Institute, Singapore) using angiograms and colour fundus photographs [13]. The graders at the CRC were masked to the clinical presentation, visual acuity and treatment arm of the patients.

The standardised diagnostic criteria used in the EVEREST study to confirm PCV diagnosis consisted of the presence of early sub-retinal focal hyperfluorescence on ICGA (within the first 6 min), and at least one of the following criteria: (1) nodular appearance of the polyp(s) on stereoscopic examination, (2) hypofluorescent halo around the nodule(s), (3) presence of a BVN, (4) pulsation of the polyp(s) on dynamic ICGA, (5) orange sub-retinal nodules on colour fundus photography that correspond to the ICGA nodules, or (6) massive sub-macular haemorrhage (≥4 disc areas in size). Angiograms of all the patients were further graded by trained retinal specialists at the CRC (CST and THL) in order to identify the features which differentiated PCV from non-PCV cases. Lesion size was determined by using a best-fit circle which was drawn around each polyp using the proprietary Heidelberg Eye Explorer software, as previously described. [13] The diameter of this circle was taken as the diameter of the polyp.

### Statistical analysis

Statistical analysis was performed using SPSS version 16.0 (SPSS Inc, Chicago, IL, USA), with p values <0.05 regarded as statistically significant. Continuous variables were compared using *t*-tests, while categorical variables were compared using the chi-square or Fisher exact test.

## Results

Of the 95 patients screened, 61 were diagnosed with PCV by the CRC and enrolled in the EVEREST study, and the remaining 34 patients were reported as screening failures. All patients were treatment naive, and only one eye from each patient was included in this study. For patients with bilateral disease, the eye with the more severe pathology was selected.

The screening failure cases (*n* = 34) were categorised into three groups: (1) PCV cases not suitable for recruitment because the location and/or lesion size of PCV did not meet the inclusion criteria (*n* = 14); (2) cases with equivocal lesions on ICGA characterised by small hyperfluorescent dots (which were typically <150 μm in diameter) (*n* = 9); and (3) cases that were definitely not PCV or ‘non-PCV’ cases, where a positive differential diagnosis was made (*n* = 11). There were no significant differences in age, sex, or laterality among patients with confirmed PCV and those with non-PCV.

The 11 cases that were identified as non-PCV included one case each of micro-aneurysms, RAP, retino-choroidal anastomosis, focal CNV, RPE window defect, and disciform scar; two cases of hyperfluorescent spots located where the choroidal vessel changed its course; and three cases of late-onset RPE staining (Table 1). A selection of these cases is described below.

**Table 1:** Demographics and features of pseudo-PCV cases

Sr. No.	Age (years)	Gender	Eye (R/L)	Diagnosis	BVN	Hypofluorescent halo	Nodularity	Orange nodule	Pulsatile	Massive submacular haemorrhage
1	76	Male	R	Late onset RPE staining	No	No	No	No	No	No
2	69	Male	L	Late onset RPE staining	No	No	No	No	No	No
3	66	Male	L	Late onset RPE staining	Yes	No	No	No	No	No
4	67	Male	R	RPE window defect	No	No	No	No	No	No
5	84	Male	R	Choroidal vascular knuckle	Yes	No	No	No	No	No
6	52	Female	L	Choroidal vascular knuckle	Yes	No	No	No	No	No
7	54	Male	R	Micro-aneurysm	No	No	No	No	No	No
8	18	Female	R	RAP	No	No	Yes (intraretinal)	No	No	No
9	62	Male	R	Retinal choroidal anastomosis	Yes	No	Yes (intraretinal)	Yes	No	No
10	69	Female	L	Focal CNV	No	Yes	No	No	No	No
11	76	Male	R	Disciform scar	No	No	No	No	No	No

*BVN* branching vascular network, *CNV* choroidal neovascularisation, *PCV* polypoidal choroidal vasculopathy, *R*/*L* right/left, *RAP* retinal angiomatous proliferation, *RPE* retinal pigment epithelium

### Cases identified as non-PCV

**Fig. 1:**
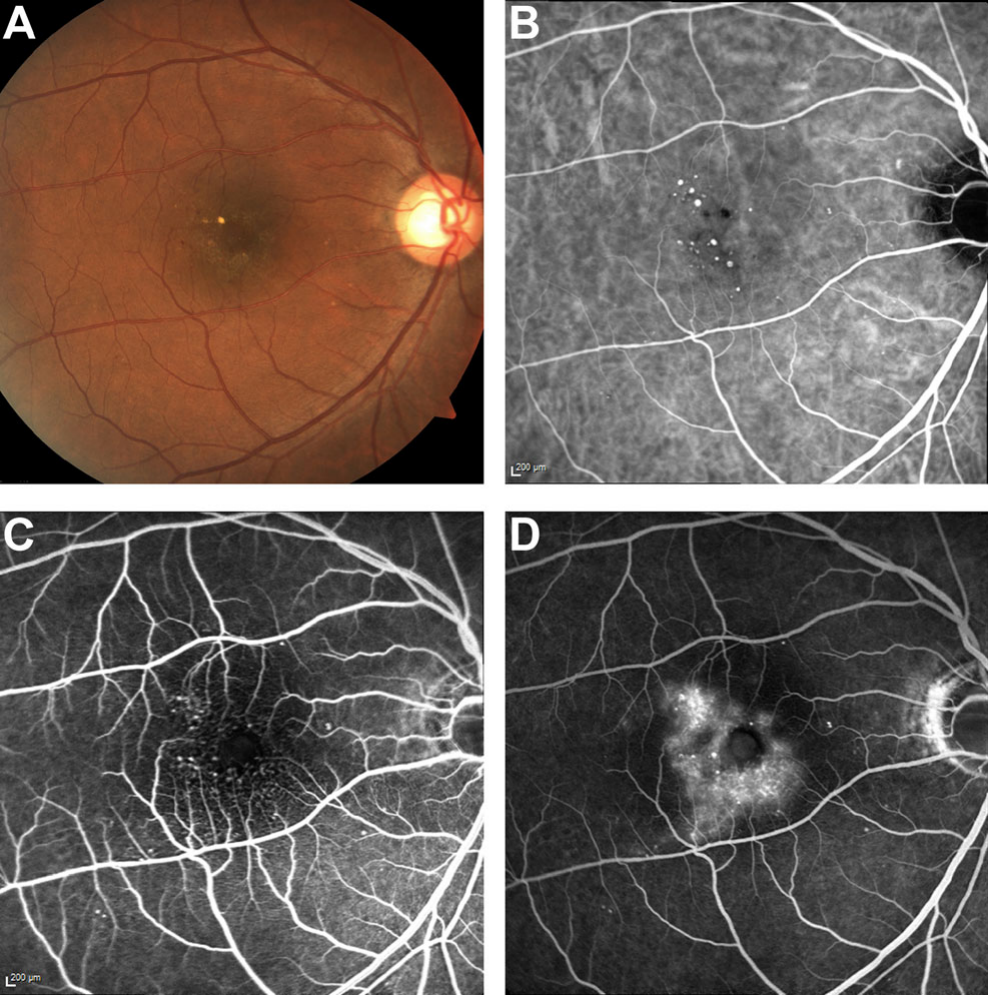
Patient with retinal microaneurysms. (**a**) Colour fundus photograph showing hard exudates with multiple small red lesions, consistent with microaneurysms; (**b**) multiple small round hyperfluorescent lesions are seen on ICGA; (**c**) foveal avascular zone irregularity on early FA, together with multiple small discrete areas of hyperfluorescence; and (**d**) late leakage from the hyperfluorescent regions, and pooling within an intraretinal cyst. *FA* fluorescein angiography, *ICG* indocyanine green angiography
- *RAP*. In this patient, focal early hyperfluorescence was observed on the ICGA image of the study eye, which suggested the possible presence of a polyp. However, dynamic ICGA showed filling of this lesion from the retinal circulation and subsequent drainage of the dye into the retinal vein superiorly. In addition, stereoscopic examination showed that the lesion was superficial. Other features, such as BVN, pulsation, orange nodules, or massive submacular haemorrhage were not observed. FA images showed leakage and pooling within intra-retinal cystoid spaces. These findings indicated the presence of RAP in the study eye (Fig. 2).

- *Microaneurysms secondary to diabetic macular oedema*. This patient manifested with hard exudates and small red lesions, which may be confused with orange nodules of PCV. On ICGA, multiple small discrete round hyperfluorescent lesions were seen. Stereoscopic examination of these lesions revealed that they were superficially located, within the retinal layers. No other features of PCV, such as BVN or hypofluorescent halo, were observed. Fluorescein angiography (FA) images revealed an irregular foveal avascular zone, telangiectasia, and late leakage from these lesions, which were diagnosed as retinal microaneurysms secondary to diabetic maculopathy (Fig. 1).

**Fig. 2:**
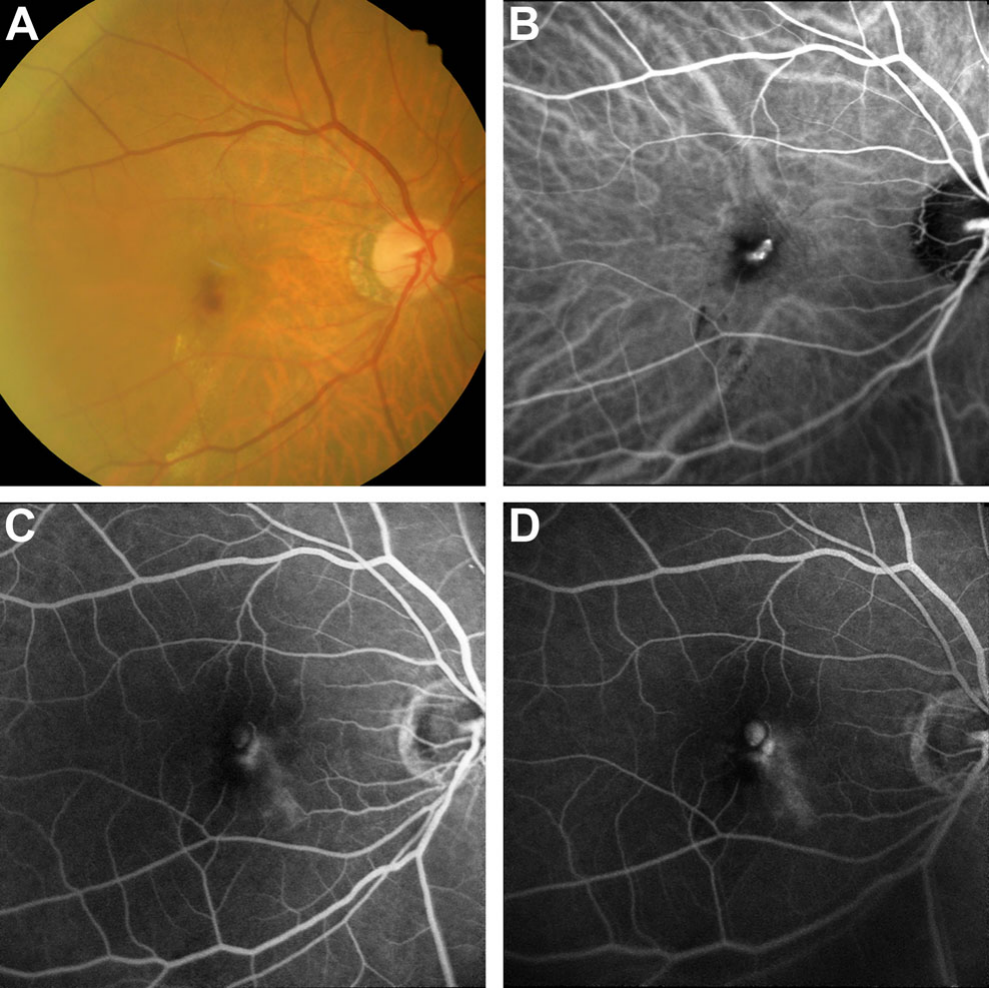
Patient with RAP. (**a**) Colour fundus photograph showing macular haemorrhage; (**b**) area of hyperfluorescence on ICGA, which resembles a polyp. However, this lesion is filled from the retinal circulation; (**c**) and (**d**) FA showing leakage and pooling of the dye within cystic spaces. *FA* fluorescein angiography, *ICG* indocyanine green angiography, *RAP* retinal angiomatous proliferation
- *Retinal pigment epithelium window defect*. Subretinal haemorrhage surrounding a CNV lesion was seen at the macula. An area of early ICGA hypefluorescence was noted along the inferotemporal arcade (Fig. 3). However, stereoscopic examination revealed that it was located in the choroid, beneath an area of RPE defect. Further examination of the dynamic ICGA showed that the area was continuous with the large choroidal vessel. In addition, other ICGA features typical of PCV were absent. Closer examination of the colour fundus photography revealed a corresponding area of chorioretinal atrophy. Likewise, a corresponding area of RPE window defect was noted on FA. This enhanced the visibility of the underlying choroidal vessel, which made it look brighter on ICGA.

**Fig. 3:**
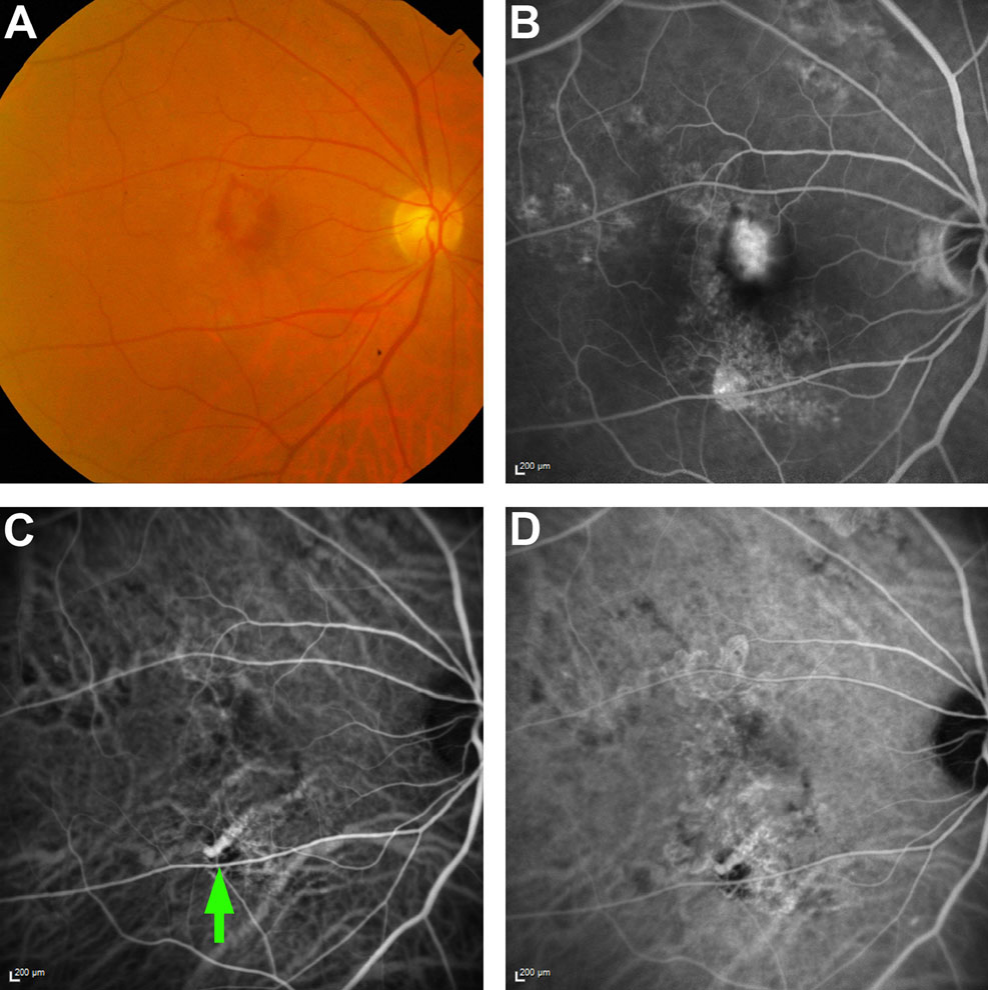
Retinal pigment epithelium window defect. (**a**) Colour fundus photography showing an area of chorioretinal atrophy inferior to the macula; (**b**) FA showing RPE window defect corresponding to the area of atrophy; (**c**) Early ICGA showing an area of hyperfluorescence corresponding to the area of atrophy. Closer examination shows that this is continuous with the underlying choroidal vessel; (**d**) Late phase ICGA showing persistence of the hyperfluorescence. *ICG* indocyanine green angiography
- *Change in course of the choroidal vessel*. This patient had subretinal haemorrhage at the edge of the macula, and examination of the ICGA showed a circular area of hyperfluorescence within the macula. However, on stereoscopic examination, it was found that the lesion was neither nodular nor raised. A closer examination of the dynamic ICGA images showed that this lesion was continuous with the underlying choroidal vessels, and occurred at a site where the choroidal vessel changed its course. The resultant increase in blood column at that location resulted in increased intensity of the ICGA hyperfluorescence, which appeared similar a polyp. Patchy chorioretinal atrophy was also observed at the macula, which enhanced visibility of the underlying choroidal vessels, thus making this region look more fluorescent on the ICGA (Fig. 4).

**Fig. 4:**
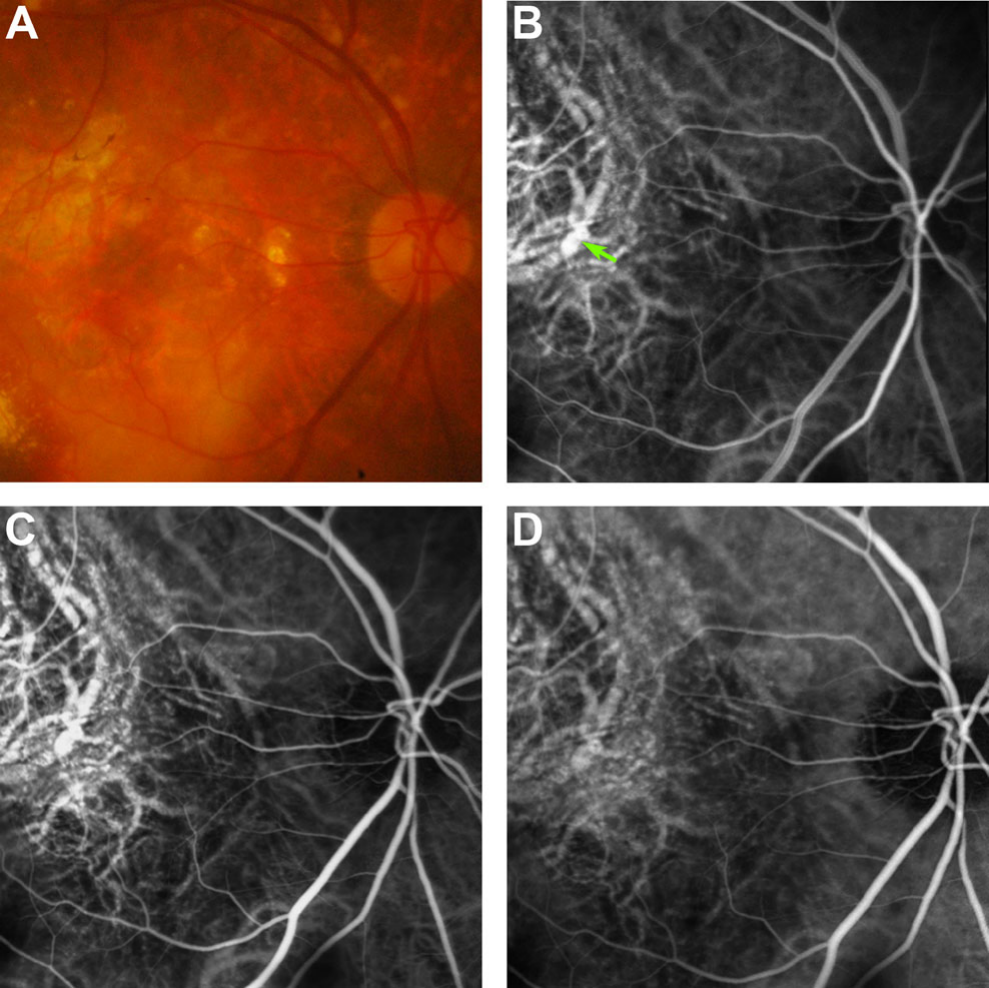
Patient with change in course of the choroidal vessel. (**a**) Colour fundus photograph showing subretinal haemorrhage inferotemporally, with regions of chorioretinal atrophy at the macula; (**b**) A ICGA demonstrating a rounded area of hyperfluorescence (*green arrow*), which does not have a hypofluorescent halo; (**c & d**) Later phases of the ICGA show that this lesion is continuous with the underlying choroidal vessel, and appears more prominent due to the area of chorioretinal atrophy. *ICG* indocyanine green angiography
- *Focal RPE staining*. Focal hyperfluorescence was noted nasal to the fovea at 10 min still frame of ICGA (Fig. 5). A separate CNV lesion was noticed in the subfoveal region, non-contiguous to the late hyperfluroescence. Examination of 1-, 3-, and 5-min still frames did not show any convincing lesion at the same location. In addition, the 20-min still frame did not show “late dye hollowing”. Stereoscopic examination showed the lesion at the level of RPE. The lesion is consistent with focal RPE staining with ICG dye.

**Fig. 5:**
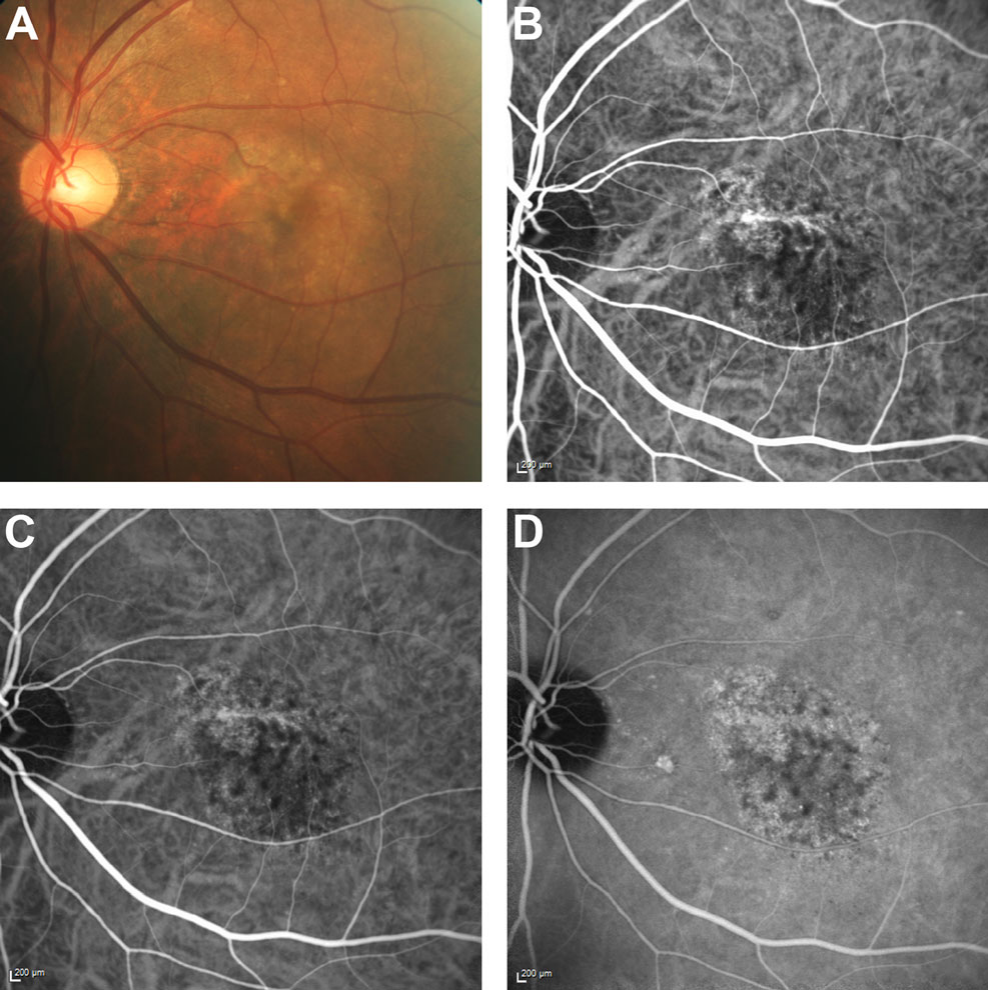
Patient with focal RPE staining. (**a**) Colour fundus photograph demonstrating a choroidal neovascular (CNV) lesion subfoveally; (**b**) Early ICGA demonstrating the network supplying the CNV lesion, but no hyperfluorescent nodule; (**c**) Mid-phase ICGA at 3 min, which still does not show any lesion resembling polyps; (**d**) ICGA at 10 min, demonstrating an area of hyperfluorescence underlying a small vessel. Correlation with the colour fundus photograph reveals that this corresponds to an area of RPE depigmentation. *FA* fluorescein angiography, *ICG* indocyanine green angiography, *RPE* retinal pigment epithelium
- *Disciform scar*. Examination of the ICGA revealed an area of early hyperfluorescence along the inferotemporal arcade. However, this lesion was not nodular and did not manifest with a surrounding halo or pulsation. In addition, there was no associated BVN. FA showed staining of the disciform scar, with no active leakage at the region of the lesion. A head-on (en-face) view of the feeder vessel of the lesion emerging from the choroidal circulation may be confused with a PCV lesion.

## Discussion

In this study, we have described and illustrated some of the conditions that were initially diagnosed as PCV at the screening stage in the EVEREST study. A detailed assessment using the standardised imaging and grading techniques allowed the investigators to distinguish conditions such as micro-aneurysm, RAP, changes in course of the choroidal vessel and RPE staining/defect from actual PCV. These conditions are often misdiagnosed as PCV because their ICGA features manifest with hyperfluorescent lesions that appear similar to polyps on single still frames. The use of stereoscopic still frames and dynamic ICGA provided additional information such as nodularity and depth of the lesion (microaneurysms, RPE window defects), presence or absence of BVN and filling pattern (RAP).

Clinically, it is important to differentiate actual PCV from non-PCV cases to ensure that patients receive appropriate and effective treatment for their condition. For example, PCV lesions can respond well to the combination of verteporfin PDT and anti-vascular endothelial growth factor (anti-VEGF), whereas RAP and micro-aneurysms respond well to anti-VEGF monotherapy [27]. Patients with focal hyperfluorescence resulting from changes in course of the choroidal vessel, focal RPE staining, and disciform scar do not require treatment if these occur in isolation. While PDT is effective in closing polyps [18], it is not without risk. Studies have reported complications following PDT, including subretinal, vitreous, and suprachoroidal hemorrhage, as well as tears and rips of the retinal pigment epithelium [28–30]. PDT is also believed to cause choroidal ischemia and thrombosis, with resultant up-regulation of VEGF production [31–34]. It is important, therefore, to distinguish PCV from non-PCV lesions, which may not require the addition of PDT to the treatment.

In a clinical trial situation, in which only patients with actual PCV may be eligible for inclusion, failure to identify patients with non-PCV at the initial presentation itself may result in some patients being recruited wrongly. This may also affect the long-term treatment outcomes and skew the study results.

Our understanding of nAMD and the wider spectrum of choroidal neovascular conditions including CNV, PCV, and RAP has increased in recent years. Advances in imaging and grading technologies have contributed to characterising these conditions accurately to aid their definitive diagnosis. Clinical experience has shown that these conditions are unique and may warrant individualised treatment for best possible outcomes [27, 35]. Data from this post-hoc analysis have shown that definitive diagnosis of PCV is possible at initial medical presentation by using ICGA together with a standardised definition for PCV diagnosis [24]. The analysis further suggests that although the presence of ICGA hyperfluorescence is the hallmark of PCV, application of diagnostic criteria, and standardised grading protocol of ICGA images are essential to prevent the misdiagnosis of polyps. ICGA has been an essential diagnostic tool for PCV for decades; however, the use of recent image grading techniques described in our analysis may help to achieve a more consistent diagnosis of PCV.

The stereo-imaging and dynamic ICGA techniques used in our analysis provided additional information for image grading and enabled differentiation of non-PCV cases from actual PCV. Stereo-imaging has been shown to assist in the differentiation of a polyp (nodular in appearance) from an RPE window defect or late RPE staining, which appears flat. By examining the location of the lesion, superficial retinal lesions such as micro-aneurysms can be differentiated from polyps, which usually occur deep beneath the RPE. In addition, the depth of the lesion and lack of nodularity is helpful in distinguishing a prominent choroidal vessel beneath an area of chorioretinal atrophy from actual polyps. Dynamic ICGA, on the other hand, can identify the difference in the pattern of blood flow between a RAP lesion and the retinal vessels.

The ICGA imaging standards, grading methods and diagnostic criteria described in the recent EVEREST study report for diagnosis of PCV can be useful in clinical practice and for future randomised controlled trials on PCV [13]. Small hyperfluorescent lesions on ICGA were excluded if these occurred in isolation (without any other diagnostic criteria of PCV) because it was difficult to determine nodularity in such small lesions. In this study, the presence of massive submacular hemorrhage with a hyperfluorescent lesion on ICGA was sufficient for the diagnosis of PCV. It was felt that a large submacular haemorrhage may mask the presence of features such as BVN, a hypofluorescent halo or the presence of an orange nodule. This decision was based on a consensus agreement of the diagnostic criteria for PCV prior to the start of this study. In addition, while submacular haemorrhages do occur in nAMD, massive macular haemorrhages are uncommon [36].

In addition to some interesting findings, our study had a few limitations. Our analysis included a small sample size. Also, we did not use optical coherence tomography (OCT) as part of the diagnostic criteria, which would have further aided in diagnosis of PCV. This study was performed in 2008, when time domain OCT was commonly used. The advent of spectral domain OCT (SD-OCT) may improve the definitive diagnosis of PCV, and is the subject of newer, ongoing studies. The patients who were diagnosed with conditions other than PCV were excluded from this prospective study. As a result, it is unknown if any of these patients may have subsequently developed PCV.

In conclusion, awareness of the conditions that may mimic PCV, and their imaging characteristics, will help to reduce the possibility of misdiagnosis. Retina specialists will, therefore, be able to provide appropriate treatment to patients with true PCV, resulting in effective management of this condition. Further studies using SD-OCT, stereo-imaging, and dynamic ICGA techniques can help develop standardised criteria for the accurate diagnosis of PCV, thus enhancing patient management.

## Electronic supplementary material

Below is the link to the electronic supplementary material.

ESM 1 (DOCX 40 kb)
