# Supplementary material for: EVEREST study report 3: diagnostic challenges of polypoidal choroidal vasculopathy. Lessons learnt from screening failures in the EVEREST study
Source: Graefes Arch Clin Exp Ophthalmol. 2016 May 3;254(10):1923–30. doi: 10.1007/s00417-016-3333-y (PMC5045476; doi:10.1007/s00417-016-3333-y)
Supplement: Supplementary file 1 — (DOCX 40 kb) [file 417_2016_3333_MOESM1_ESM.docx]

**SUPPLEMENTARY FILES**

**Supplementary S1: The EVEREST study group**

**List of group members involved in the EVEREST study:**

| **NAME** | **INSTITUTION** | **COUNTRY** |
| --- | --- | --- |
| Timothy Lai | Chinese University of Hong Kong  Hong Kong Hong Kong NIL  China Hong Kong | China |
| Adrian Koh | Tan Tock Seng Hospital  Singapore Singapore 308433  Singapore | Singapore |
| Paisan Ruamviboonsuk | Rajavithi Hospital  Bangkok Bangkok 10400  Thailand | Thailand |
| Won Ki Lee | The Catholic University of Korea  Seoul St.Mary's hospital  Seocho Gu Seoul 137-701  Republic of Korea | Republic of Korea |
| HaKyoung Kim | Kangnam Sacred Heart Hospital  Youngdeungpo Gu Seoul 150-  030, Republic of Korea | Republic of Korea |
| Shih-Jen Chen | Taipei Veterans General Hospital  Teipei Taiwan, ROC 112  Taiwan | Taiwan |
| Lee-Jen Chen | MacKay Memorial Hospital  Teipei Taiwan  Taiwan | Taiwan |
